# Recent High-Resolution Structures of Amyloids Involved in Neurodegenerative Diseases

**DOI:** 10.3389/fnagi.2021.782617

**Published:** 2021-11-19

**Authors:** Rodrigo Diaz-Espinoza

**Affiliations:** Departamento de Biología, Facultad de Química y Biología, Universidad de Santiago de Chile, Santiago, Chile

**Keywords:** neurodegenerative diseases, misfolding, amyloid, structure, Aβ, Tau, α-Sn

## Abstract

Amyloids are highly ordered aggregates composed of proteins or peptides. They are involved in several pathologies, including hallmark neurodegenerative disorders such as Alzheimer’s (AD) and Parkinson’s (PD). Individuals affected by these diseases accumulate in their brains amyloids inclusions composed of misfolded forms of a peptide (Aβ) and a protein (Tau) in AD and α-synuclein protein (α-Sn) in PD. Tau and α-Sn aggregates are also present in other neurodegenerative diseases. The insoluble nature and heterogeneity of amyloids have hampered their study at the molecular level. However, the use of solid state NMR and Cryogenic-electron microscopy along with fine-tuned modulation of the aggregation *in vitro* and improved isolation methods of brain-derived amyloids has allowed the elucidation of these elusive conformations at high resolution. In this work, we review the latest progress on the recent amyloid structures reported for Aβ, Tau, and α-Sn. The two-fold symmetry emerges as a convergent feature in the tridimensional arrangement of the protofilaments in the fibrillary structure of these pathological amyloids, with many of them exhibiting a Greek-key topology as part of their overall architecture. These specific features can serve as novel guides to seek potential molecular targets in drug design efforts.

## Introduction

The amyloid state of proteins is nowadays recognized as a convergent conformation that is accessible to most proteins and peptides under the appropriate conditions (Hartl and Hayer-Hartl, 2009). However, the proteinaceous nature of amyloids was recognized decades after their original descriptions (Kyle, 2001). Since then, amyloid deposits have been identified in different human pathologies and also in many organisms as functional assemblies (Chiti and Dobson, 2017). These aggregates are relevant pathological agents in neurodegenerative disorders such as AD and PD where they accumulate in the brain as inclusions with disease-dependent morphological features and location (Terry, 1963; Spillantini et al., 1997; Selkoe and Hardy, 2016). In AD, the affected individuals have two types of amyloid deposits that are composed of aggregated Aβ or misfolded Tau, whereas in PD misfolded α-Sn forms intracellular aggregates. Their specific pathological roles constitute active areas of research. However, their peculiar structural features have imposed important challenges to their study at the molecular level. Since these are unique pathological agents, elucidating their structures is crucial to design novel therapeutic approaches. We will review here the three iconic amyloids involved in neurodegenerative diseases along with their recently solved high-resolution structures.

## Overview of Amyloid Structure

Amyloids are highly ordered aggregates stabilized by a beta-sheet core (Greenwald and Riek, 2010). Misfolded proteins or peptides are arranged in the beta-sheet in an intermolecular fashion through a hydrogen-bonded network in which the strands are transversally disposed with respect to the growing axis of the amyloid fibril, creating an overall “cross-beta” pattern (Riek, 2017). Beta-sheets can be arranged in a parallel or anti-parallel manner. Interestingly, regardless of the specific sequence all amyloids exhibit a similar overall architecture. This is indicative of a convergent misfolding pathway (Hartl and Hayer-Hartl, 2009). Moreover, amyloids are formed by the self-assembly of not only proteins but also small peptides and even single aromatic amino acids, indicating that the amyloid fold may be structurally and universally encrypted in the hydrocarbon chain of polypeptides (Balbach et al., 2000; Dobson, 2004; Adler-Abramovich et al., 2012). Still, the type and ordering of the amino acids within a given sequence is recognized to have a significant role in the aggregation propensity, with hydrophobic residues being the main contributors to the stability of the amyloid fold (Gazit, 2002; Hall et al., 2005; Greenwald and Riek, 2010). Despite their structural convergence, amyloids can still exhibit structural diversity at the molecular level (Gallardo et al., 2020). Such variations can be dependent on the specific polypeptide sequence as well as on the size.

Although amyloids are in general very stable, the non-covalent nature of the intermolecular interactions makes amyloid fibrils relatively fragile, which can fragment into smaller fibrils when subjected to mechanical or chemical stresses (Xue et al., 2010). Therefore, amyloids suffer from an inherent heterogeneity with respect to size distribution (Morales et al., 2016; Eisenberg and Sawaya, 2017). Such heterogeneity and their often-low solubility has made their structural characterization extremely challenging using classical structural biology techniques, let alone high-resolution approaches (Diaz-Espinoza and Soto, 2012; Fändrich et al., 2018). The first high-resolution amyloid structures were achieved using small peptides that formed amyloid-like structures when subjected to controlled dehydration (Nelson et al., 2005; Sawaya et al., 2007). The fine-tuning of experimental approaches aimed at increasing the homogeneity of the samples as well as implementing and combining novel and refined techniques such as sold-state NMR (ssNMR) and Cryogenic-Electron Microscopy (Cryo-EM) have allowed to obtain high-resolution structures of several amyloids, including those formed by Aβ, Tau, and α-Sn.

## Aβ

Aβ are peptides of varying sizes released upon proteolytic cleavage of a membrane protein (amyloid precursor protein) (Kang et al., 1987). Some of these peptides can aggregate into fibrillary species, which then accumulates as plaques in the brain (Glenner and Wong, 1984; Selkoe and Hardy, 2016). These plaques typically contain Aβ fragments 1–40 (Aβ_40_) and 1–42 (Aβ_42_) among other components (Roher et al., 1993). Cellular toxicity associated to misfolded Aβ proceeds through diverse mechanisms including microglia-mediated localized inflammation around the plaques, impaired synapsis, membrane disruptions induced by Aβ oligomeric species, metals imbalance, etc (Cavallucci et al., 2012). Moreover, Aβ fibrils and oligomers (Aβ_40_ and Aβ_42_) prepared *in vitro* using chemically synthesized or recombinant Aβ peptide display dose-dependent cytotoxicity in cell cultures of diverse neuronal lines (Zagorski et al., 1999; Finder et al., 2010). The *in vitro* assembly of synthetic Aβ into amyloids has been characterized extensively in the literature and has enabled access to molecular details of Aβ aggregation, which may partially resemble the *in vivo* mechanisms during plaque formation (Caughey and Lansbury, 2003). Aβ_42_ is more abundant in the plaques than Aβ_40_ and hence it has been studied in greater detailed (Roher et al., 1993). The assembly into amyloids *in vitro* from monomeric Aβ_42_ is a sequential nucleation-polymerization (NP) process that involves the emergence of nucleating oligomers followed by the formation of small fibrillary species or proto-fibrils, which then associate to form mature fibrils (Hardy and Higgins, 1992; Chen et al., 2017). The plaques in AD contain mature-like Aβ_42_ fibrils and hence these fibrils can serve as an *in vitro* model for their study. Aβ_42_ fibrils subjected to mechanical disruption can give rise to small fragments that can act as seeds of Aβ_42_ polymerization, accelerating the formation of mature fibrils. This seeding mechanism along with the NP process is a general trait of amyloids (Jarrett and Lansbury, 1993; Ke et al., 2020).

**FIGURE 1:**
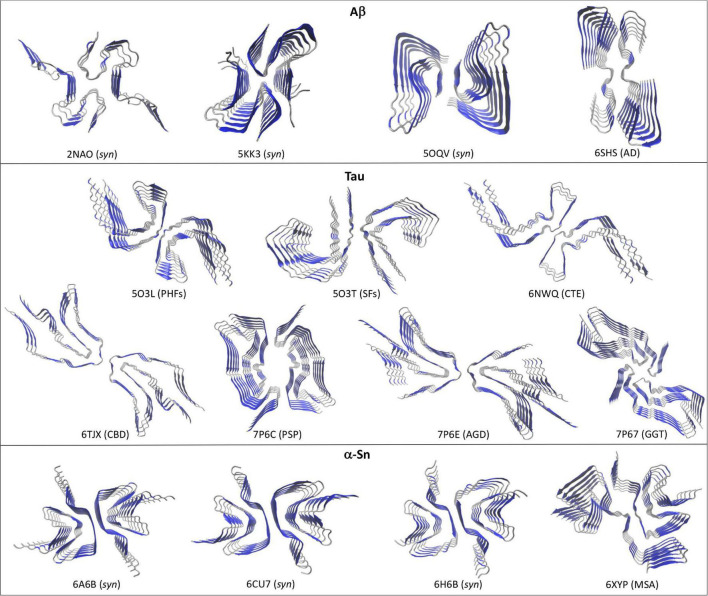
Different high-resolution structures of Aβ, Tau, and α-Sn in the amyloid state. Top views are shown to highlight the two-fold symmetry arrangement of the protofilaments. Aβ: 2NAO, 5KK3, and 5OQV are synthetic Aβ_42_ fibrils (*syn*); 6SHS are brain-derived Type II Aβ_40_ fibrils from AD. Tau: 5O3L and 5O3T are respectively, *ex vivo* PHFs and SFs from AD; 6NWQ, 6TJX, 7P6C, 7P6E, and 7P67 are *ex vivo* Type II filaments from CTE, CBD, PSP, AGD, and GGT, respectively. α-Sn: 6A6B, 6CU7, and 6H6B are synthetic fibrils (*syn*); 6XYP are brain-derived Type I fibrils from MSA. Beta-sheets are depicted in blue and coils and α-helices in silver.

Two recent works have produced the first structures of full length Aβ_42_ in the amyloid state and at atomic resolution using ssNMR combined with molecular dynamics (MD; Colvin et al., 2016; Wälti et al., 2016). Wälti et al. (2016) showed that serially seeding the *in vitro* aggregation of synthetic Aβ_42_ along with fine-tuning the solution conditions can produce highly homogenous Aβ_42_ fibrils, with the resulting fibrils being recognized by plaque-specific antibodies. Colvin et al. (2016) grew Aβ_42_ fibrils overnight at cooler temperatures without agitation to produce the homogenous fibrils. Interestingly, both approaches yielded very similar structures (Figure 1, pdb codes 2NAO and 5KK3). The amyloids are arranged by a two-fold symmetry, in which two protofilaments are stabilized mainly by lateral contacts. Each Aβ_42_ monomer is stacked in a parallel, in-register fashion along the fibril axis. In both models the Aβ_42_ N-terminal region (1–14) does not make specific contacts with the amyloid core probably due to its flexible and less hydrophobic nature. In a later work, Gremer et al. (2017) combined data from ssNMR with Cryo-EM to provide a novel structure for Aβ_42_ in a fibrillary state. Homogenous fibrils were obtained by growing Aβ_42_ at low pH in a solution containing a small fraction of acetonitrile and trifluoroacetic acid. The resulting fibrils were similarly toxic to PC12 rat brain cells as fibrils grown at neutral pH. Considering the fibrillary nature of this structure, the resulting model exhibited several significant differences with the previous models (Figure 1, pdb code 5OQV). First, the N-terminal region (1–15) appeared as part of the amyloid arrangement and engaged in specific contacts including intermolecular (^1^Asp-^28^Lys) and intramolecular salt bridges (^5^Arg-^7^Asp, ^6^His-^13^His-^11^Glu). Secondly, the C-terminal region (38–42) makes direct intermolecular, inter-subunit hydrophobic contacts that appears as key in stabilizing the two-fold symmetry, whereas in the previous models these residues extend outward the amyloid core. Interestingly, Aβ_40_ lacks the last two of these C-terminal residues and peptides containing the full C-terminal region (sequence GGVVIA) spontaneously self-assembly into amyloids *in vitro*, which may suggest that this fibril model may be capturing a key feature of plaque-like Aβ_42_ fibrils (Sawaya et al., 2007). Despite these differences, all the structures show a left-handed twist with a general two-fold symmetry formed by two interacting protofilaments.

Although high-resolution structures of full length Aβ_42_ fibrils isolated from diseased brains (*ex vivo*) are not yet available, fibrillary structures of brain-derived Aβ_40_ fibrils from an AD-affected patient were recently solved using Cryo-EM (Kollmer et al., 2019). These structures differed significantly from those obtained with synthetic Aβ_42_ fibrils in that they showed a right-handed twist, different stabilizing hydrophobic contacts and different arrangements of the protofilaments. A single protofilament fibrillary arrangement was dominant in the sample (Type I), in which two stacks of Aβ_40_ peptides are stabilized by hydrophobic contacts and arranged symmetrically. Additional fibrils were also observed in which two (Type II) or three (Type III) Type I protofilaments interact through lateral contacts mediated by two salt bridges (^3^Glu-^5^Arg), with Type II filaments showing a two-fold symmetry (Figure 1, pdb code 6H6S). It remains to be seen whether these structural features are exclusive of *ex vivo* Aβ_40_ fibrils or they rather represent a convergent trait of brain-derived Aβ fibrils.

## Tau

In addition to Aβ_42_ plaques, AD-affected brains can also contain intracellular inclusions composed of misfolded Tau protein (Terry, 1963; Lee et al., 1991). These Tau filaments give rise to neurofibrillary lesions, which can accumulate in different areas of the affected brains, including neocortex and limbic system, among others. In the case of AD, two types of Tau inclusions are commonly: paired helical filaments (PHFs) and straight filaments (SFs; Kidd, 1963; Crowther, 1991). Tau-associated inclusions are not an exclusive feature of AD and similar depositions can be found in other neurodegenerative disorders such as Pick’s disease (PiD), chronic traumatic encephalopathy (CTE), corticobasal degeneration (CBD), progressive supranuclear palsy (PSP), argyrophilic grain disease (AGD) and globular glial tauopathy (GGT), among others (Crowther and Goedert, 2000; Ghetti et al., 2015).

**FIGURE 2:**
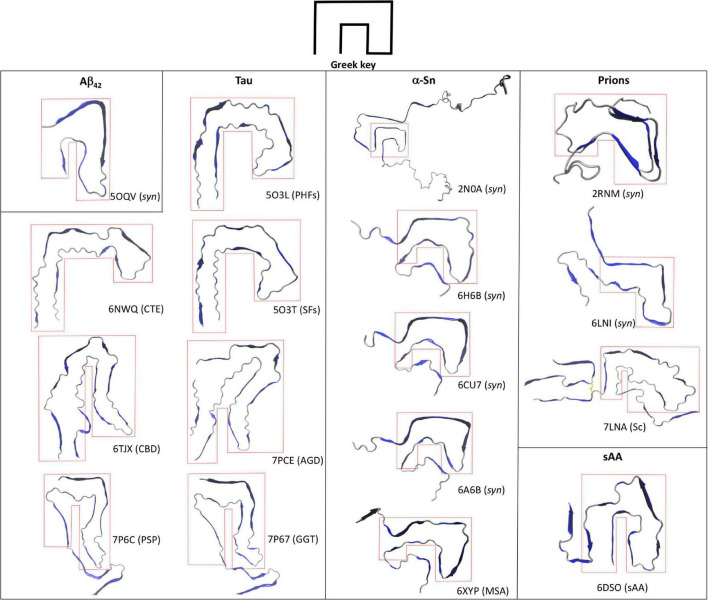
Greek-key topology in the amyloid fold. Top views of the single chains of several amyloid structures of Aβ_42_, Tau, and α-Sn are shown along with their associated disease or synthetic nature (*syn*). Synthetic HET prion (2RNM), human recombinant prion protein in the amyloid state (6LNI), infectious hamster brain-derived prion protein (7LNA), and mouse spleen-derived acute phase protein serum amyloid A1 (6DSO) are depicted for comparison. The enclosing red dotted lines highlight the Greek-key topology in each structure.

Contrary to Aβ, Tau is a constitutively expressed protein involved in cytoskeleton homeostasis through direct stabilizing interactions with microtubules (Desai and Mitchison, 1997). At the structural level, Tau is an intrinsically disorder protein that can be found in six isoforms with sequence lengths varying from 352 to 441 amino acids (Goedert et al., 1989; Schweers et al., 1994). The details of Tau aggregation *in vivo* are intricate and different mechanisms have been proposed to contribute, including post-translational modifications such as hyperphosphorylation, glycosylation and truncation, ligand interactions, dimerization events, etc (Goedert et al., 1992; Alquezar et al., 2020). Such complexity has been very difficult to recreate *in vitro* and hence synthetic Tau fibrils that fully mimic PHFs and SFs is an ongoing challenge. However, alternative approaches that rely on isolating Tau amyloid inclusions at high homogeneity from diseased brains has proven a valuable tool for structural studies. In fact, high resolution structures of Tau PHFs and SFs isolated from an AD-affected brain have been recently solved using Cryo-EM (Fitzpatrick et al., 2017). Fitzpatrick et al. (2017) isolated and purified sarkosyl-insoluble fractions that were enriched in PHFs and SFs, which were then used to obtain high resolution structures of both filaments (Figure 1). Both PHFs and SFs showed a two-fold symmetry similar to that observed with *in vitro*-prepared Aβ_42_ fibrils, in which Tau monomers are stacked through parallel beta-sheets with each molecule containing eight beta strands alternated with loop regions, forming two protofilaments running along the fibril axis. Interestingly, each Tau protofilament is arranged in a clear Greek-like key pattern (Figure 2). Compared to Aβ, only one of all the solved Aβ fibril structures showed this topology and with a much less organized fashion. Although Tau is not particularly hydrophobic as Aβ_42_, the main intramolecular hydrophobic contacts reside precisely at the end of the end hinge region of the Greek-key fold, which may help stabilize this particular shape. Intermolecular contacts between the two protofilaments in PHFs involve H-bonds among residues ^332^PGGGQ^336^ in an anti-parallel fashion. SFs intermolecular interactions are more asymmetrical, with contacts between region ^321^KCGS^324^ and ^313^VDLSK^317^, though the specific types of stabilizing interactions are not yet clear. Using a similar approach the same group have elucidated high resolution structures of brain-derived Tau amyloids from patients affected with PiD, CTE, CBD, PSP, GPT, or GGT (Falcon et al., 2018, 2019; Zhang et al., 2020; Shi et al., 2021). As observed with brain-derived Aβ_40_ amyloids, heterogeneous arrangements of the protofilaments were evident in several of the structures, particularly in those from CBD, CTE, PSP, and GGT. Still, the two-fold symmetry emerged again as a common feature in Type I and II filaments in CTE, and Type II filaments from CBD, PSP, AGD, and GGT, whereas single protofilament arrangements included Tau from PiD, PSP and Type I filaments from CBD, PSP, AGD, and GGT (Figure 1). Regardless of the disease, most Tau filaments have the Greek-key topology as a general feature, including Type I and II filaments from CTE, CBD, PSP, and AGD and in a less organized fashion the single protofilaments from PSP and Type I and II filaments from GGT (Figure 2).

## α-Sn

One of the hallmark and specific pathological features of PD is the formation of intracellular depositions called Lewy bodies (LB) and Lewy neurites (LN) in nerve cells (Yves, 1991). These inclusions are mainly composed of an abnormally folded α-Sn protein in a fibrillary state (Spillantini et al., 1998b) and hence the intracellular accumulation of fibrillary α-Sn is recognized as a pathological condition. Fibrils prepared *in vitro* using recombinant α-Sn can seed the formation of LB- and LN-like inclusions in cell culture models, reinforcing the role of α-Sn in PD (Luk et al., 2009; Volpicelli-Daley et al., 2011). Furthermore, intracerebral inoculation of these synthetic fibrils in wild-type mice can not only seed the aggregation of native brain α-Sn but also initiate a PD-like pathology in the mice (Luk et al., 2012). Therefore, these synthetic fibrils have served as important models for molecular studies.

Several high-resolution structures of synthetic α-Sn fibrils have been reported in the last years. Tuttle et al. (2016) prepared *in vitro* recombinant α-Sn fibrils (full length) that seeded in a dose-dependent manner the aggregation of α-Sn in primary hippocampal neuronal cells and also produced toxicity to the same cells at high protein concentrations. A high-resolution structure of a single α-Sn protofilament was then obtained using ssNMR, electron and X-ray fiber diffraction (Figure 1, pdb code 2NAO). Based on this structure, the amyloid Greek-key pattern was proposed for the first time (Figure 2). Interestingly, for α-Sn this topology exhibits specific intramolecular interactions that appear as poised for its overall stabilization, including a combination of hydrophobic contacts (^88^Ile-^90^Ala-^94^Phe), a salt bridge (^46^Glu-^80^Lys), and a H-bond (^79^Gln-^90^QAla). In a later work, Guerrero-Ferreira et al. (2018) reported the high-resolution structure of α-Sn in a fibrillary state by using Cryo-EM (Figure 1, pdb code 6H6B). They showed that a truncated recombinant α-Sn (residues 1–121) was the only preparation to yield homogenous fibrils upon self-assembly *in vitro*, as confirmed by EM. This truncated α-Sn is known to be highly prone to form amyloids *in vitro* and has been observed to naturally emerge in cells (Crowther et al., 1998; Li et al., 2005). The protofilaments are arranged in a two-fold symmetry (Figure 1). As observed with Tau filaments, α-Sn monomers are stacked onto the amyloid fold with eight beta strands alternated with loops in each of the protofilaments. The interface between both protofilaments is dominated by intermolecular hydrophobic interactions (^53^Ala-^53^Ala and ^55^Val-^51^Gly) and salt bridges (^50^His-^57^Glu). From the six familial mutations described for α-Sn, four occur directly in this interface, which suggest that the two-fold symmetry may have a key role in pathogenesis. The Greek-key pattern is again evident in the overall structure (Figure 2). Although the final Cryo-EM model was built using the structure from Tuttle et al. (2016) major refinements were needed for the protofilaments structure, which is indicative of structural differences. The corresponding interacting strands at the interface are positioned differently in the parent model since only a single protofilament was solved. Moreover, several key residues of the interface such as ^53^Ala are positioned inward. Two additional Cryo-EM structures for α-Sn fibrils were also reported in the same year (Figure 1, pdb codes 6A6B and 6CU7). These structures were obtained using full length recombinant α-Sn that upon *in vitro* aggregation was able to seed the misfolding of endogenous α-Sn in rat primary neurons (Li Y. et al., 2018) or HEK 293T cells (Li B. et al., 2018). Both preparations were toxic to different cell lines in a dose-dependent manner. The solved structures were very similar to that reported by Guerrero-Ferreira et al. (2018) showing again a Greek-key pattern (Figure 2). The ^53^Ala-^53^Ala interaction appears again as key for the stability of the protofilaments interface. Interestingly, an intramolecular hydrophobic contact (^70^Val-^66^Val) within the hinge of the Greek key is present in all of the reported structures, suggesting this interaction may be pivotal for stabilizing the topology. In a later work, Ni et al. (2019) reported several Cryo-EM structures using recombinant α-Sn with different truncations. The Greek-key topology was again evident in all of the structures, showing that this pattern is still maintained upon α-Sn truncation. The fact that all these synthetic fibrils exhibited similar overall structures is interesting, with a very similar overall two-fold symmetry, Greek-key topology and interface interactions.

Misfolded α-Sn forms are also present in other neurodegenerative diseases such as multiple system atrophy (MSA; Spillantini et al., 1998a). Schweighauser et al. (2020) recently reported the first high-resolution structure of brain-derived α-Sn fibrils using Cryo-EM, from a patient affected by MSA. As observed with *ex vivo* fibrillary structures of Aβ_40_ and Tau, the α-Sn protofilaments are arranged in different fashions that in this case was also patient-dependent. The described filament types (I and II) showed a very similar two-protofilament overall arrangement but organized without full symmetry (Figure 1, pdb code 6XYP). Despite the low two-fold symmetry, the Greek-key topology of each protofilament is in general remarkably similar to those observed with synthetic α-Sn fibrils (Figure 2).

## Conclusion

Advances in state-of-the-art experimental techniques such as ssNMR and Cryo-EM have enabled access to the molecular details of different amyloid assemblies. By using these techniques in combination with reducing size and conformational heterogeneity of the aggregates as well as improving isolation methods of brain-derived amyloids, solving high-resolution structures of amyloids formed by synthetic protein or *ex vivo* material of Aβ_42_, Tau, and α-Sn has became feasible. Analysis of the structures shows that at the molecular level, amyloid structures can be heterogeneous, with a single primary sequence yielding different conformations and/or protofilaments arrangements in a disease-, sample- and in some cases patient-dependent manner. Still, the fibrillary states of these proteins show that two interacting protofilaments appear as a common amyloid arrangement emerging beyond the classical cross-beta pattern. In addition, the Greek-key topology can be observed in several of the structures though this is not as a convergent feature as the two-fold symmetry. In most of the structures reviewed, the hinge of this topology appears as the minimal and ubiquitous feature, which is stabilized mainly by hydrophobic interactions. Greek-key motifs are common in proteins that have beta-barrels and beta-sandwiches, which suggests that beta-sheets are a requirement for this fold (Zhang and Kim, 2000). Interestingly, high-resolution structures of amyloids prepared with synthetic prion proteins also exhibit architectures that resemble a Greek key (Figure 2; Wasmer et al., 2008; Wang et al., 2020). Furthermore, the same topology is described in a recently reported Cryo-EM structure of a mammalian prion isolated directly from scrapie-infected (Sc) brains and can be also observed in the amyloid fibril structure of acute phase protein serum amyloid A1 involved in systemic amyloidosis (sAA; Liberta et al., 2019; Kraus et al., 2021). Still, the fact that this topology is not observed in all of the samples serves as evidence of the different amyloid conformations that even a single primary sequence can reach. Contrary to the less complex and more homogeneous amyloid structures obtained with small peptides, all these unique structural arrangements may be only accessible for proteins or large peptides, which by combining beta strands with flexible loop-like regions can then reach more intricate amyloid conformations. Rational design of drugs aimed at disrupting the protofilament interface and/or the Greek-key fold may hold promise for novel therapeutic approaches. For a given protein, however, interactions stabilizing these arrangements show great variability depending not only on the specific disease but also on the type of sample utilized (*ex vivo* fibrils, synthetic fibrils, full-length, or different fragments, etc.). Therefore, each of these models can serve as potential targets in drug design efforts for exploring and validating their biological relevance in each pathology.

## Author Contributions

RD-E conceived, wrote, revised, and approved the manuscript.

## Conflict of Interest

The author declares that the research was conducted in the absence of any commercial or financial relationships that could be construed as a potential conflict of interest.

## Publisher’s Note

All claims expressed in this article are solely those of the authors and do not necessarily represent those of their affiliated organizations, or those of the publisher, the editors and the reviewers. Any product that may be evaluated in this article, or claim that may be made by its manufacturer, is not guaranteed or endorsed by the publisher.
